# LRG1 as a Potential Therapeutic Target in Atherosclerosis: Mechanistic Basis and Current Evidence

**DOI:** 10.3390/cells15100932

**Published:** 2026-05-19

**Authors:** Jianan Wu, Xia Yi, Lanlan Wang, Kaixuan Yang, Minghuan Liu, Jiawei Song, Zenghui Yue

**Affiliations:** College of Acupuncture-Moxibustion, Tuina and Rehabilitation, Hunan University of Chinese Medicine, Changsha 410208, China; wja1224@stu.hnucm.edu.cn (J.W.);

**Keywords:** atherosclerosis, LRG1, vascular inflammation, endothelial dysfunction, therapeutic target

## Abstract

**Highlights:**

**What are the main findings?**

- LRG1 is closely associated with inflammatory amplification, endothelial dysfunction, aberrant angiogenesis, and extracellular matrix remodeling in atherosclerosis.
- Current evidence from clinical studies, lesional observations, and experimental models supports LRG1 as a candidate molecule with potential relevance to plaque progression and structural evolution.

**What are the implications of the main findings?**

- LRG1 may represent a mechanistically relevant link between vascular inflammation and plaque remodeling in atherosclerosis.
- Targeting LRG1 may offer a potential translational strategy for atherosclerosis, although further validation is still required.

**Abstract:**

Atherosclerosis (AS) is a chronic inflammatory disease of large arteries. It underlies many cardiovascular disorders, including coronary artery disease, myocardial infarction, stroke, and peripheral arterial disease. Current therapies have improved outcomes, especially lipid-lowering, antithrombotic, and anti-inflammatory treatments. Yet residual cardiovascular risk remains, and new molecular targets are still needed. Leucine-rich α-2-glycoprotein 1 (LRG1) is an inflammation-inducible secreted glycoprotein. It has drawn attention because it is linked to pathological angiogenesis, vascular dysfunction, tissue remodeling, and fibrosis. Recent studies indicate that LRG1 is related to AS at several levels. These include circulating clinical associations, plaque localization, and experimental models. In AS, LRG1 may not simply act as a biomarker. It may promote macrophage pro-inflammatory polarization, disturb endothelial homeostasis, support abnormal angiogenesis, and influence extracellular matrix remodeling and plaque structural change. This review examines the biological features of LRG1 and the current evidence connecting it with AS. It also discusses possible mechanisms, therapeutic feasibility, and current limitations. Overall, LRG1 appears to be a promising but still incompletely validated candidate target in AS.

## 1. Introduction

Atherosclerosis (AS) is a chronic inflammatory disease of large arteries. It is the pathological basis of several cardiovascular diseases, including coronary heart disease, myocardial infarction, ischemic stroke, and peripheral arterial disease [1,2]. The pathogenesis of AS is complex. It can be broadly divided into initiation, progression, and complications. Even the initiation stage involves several linked events, such as lipid deposition, inflammation, and endothelial dysfunction [1]. Innate and adaptive immune responses also participate in AS development and progression [3]. Lipid-lowering therapy remains a central strategy for patients with AS. It reduces the risk of recurrent cardiovascular events. But effective cholesterol-lowering therapies do not completely eliminate cardiovascular risk. Residual inflammatory risk remains a major and largely untreated problem [4]. A higher inflammatory burden is associated with a higher risk of adverse cardiovascular events [5]. These findings suggest that AS cannot be managed only by lipid modification [6]. Chronic low-grade inflammation is deeply involved in plaque formation, progression, rupture, and thrombosis. Cytokines, chemokines, and acute-phase reactants are important mediators in this process [7]. Therefore, additional molecular nodes involved in AS pathogenesis still need to be identified and targeted.

Leucine-rich alpha-2 glycoprotein 1 (LRG1) is an important member of the leucine-rich repeat (LRR) protein family and acts as a multifunctional pathogenic signaling molecule in a variety of diseases [8]. LRG1 exerts many of its biological effects through interactions with receptor proteins. It is closely related to pathological angiogenesis and fibrosis, especially through transforming growth factor-beta (TGF-β)-related signaling. Recent studies have reported abnormal LRG1 expression in cancers, cardiovascular diseases, diabetes, autoimmune diseases, inflammatory disorders, infectious diseases, and degenerative diseases [9]. Its function appears to vary across disease contexts. One study further suggests that LRG1 may serve as a novel biomarker for large-vessel vasculitis [10]. LRG1 can also promote the development of cardiovascular diseases by modulating the TGF-β and SMAD1/5/8 signaling pathways in endothelial cells, thereby influencing endothelial dysfunction and inflammation. Moreover, LRG1 is an important contributor to the occurrence and progression of complications associated with atherosclerotic plaques and may therefore represent a potential therapeutic target [11]. Although knockout of the *LRG1* gene or the use of anti-LRG1 neutralizing antibodies can slow the progression of AS in animal models, its specific roles at different stages of AS and its feasibility as a therapeutic target have not yet been systematically integrated [12]. Recent reviews have summarized the gene regulation, structural features, receptor interactions, disease-related functions, and therapeutic potential of LRG1 [9,13]. But the evidence related to AS remains fragmented. It is still unclear how LRG1 links systemic clinical findings with local plaque changes. The roles in vascular inflammation, endothelial injury, abnormal angiogenesis, extracellular matrix remodeling, and plaque evolution also require further synthesis. This review summarizes the biological characteristics of LRG1 and the evidence linking it to AS. We further discuss its possible mechanisms, therapeutic potential, and current limitations. This synthesis focusing on disease may help clarify the role and translational value of LRG1 in AS.

## 2. Vascular Pathophysiological Significance of LRG1

LRG1 is a secreted member of the LRR protein family [13]. It was first identified in human serum in 1977 and is a serum glycoprotein present at a concentration of 10–50 μg/mL, produced mainly by hepatocytes [14]. Subsequent studies have shown that LRG1 is not merely present in the circulation but also displays clearly inducible expression under a variety of disease conditions. Elevated LRG1 levels have been observed in many tumors and inflammatory diseases, prompting researchers to propose that it may serve as a biomarker for these conditions. In parallel, one study found that *LRG1* transcription is upregulated in myeloid cells during granulopoiesis, suggesting that it may play a role in myeloid cell maturation [15]. In addition to hepatic secretion, LRG1 can also be locally secreted in other organs and induced within lesions, where it acts through autocrine or paracrine mechanisms. Thus, LRG1 has not only humoral diagnostic value but also the potential to participate directly in local pathological processes. Preclinical and clinical studies suggest that LRG1 may contribute to the pathogenesis of diabetic vascular complications, such as diabetic retinopathy and diabetic nephropathy, by modulating the pro-angiogenic and pro-fibrotic TGF-β signaling pathway [16]. In addition, LRG1 expression can be detected both in disease specimens and in patients’ blood, with relatively stable specificity and sensitivity, which is important for disease diagnosis and prognosis [17]. Taken together, considering its molecular origin, pattern of expression, and disease-inducible characteristics, LRG1 should no longer be regarded simply as a background serum protein; rather, it should be viewed as a secreted molecule that can be activated by inflammatory and pathological stimuli and may participate in local tissue responses, thereby conferring pathophysiological significance in vascular disease.

On this basis, evidence suggests that LRG1 is not merely passively elevated during disease states, but may actively participate in the regulation of inflammation and abnormal vascular responses. One study showed that LRG1 is induced by the inflammatory microenvironment; specifically, the inflammatory cytokine interleukin-6 (IL-6) activates *LRG1* transcription through signal transducer and activator of transcription 3 (STAT3) phosphorylation, resulting in vascular instability. This finding suggests that LRG1 is positioned at a key node linking inflammatory signaling to abnormal vascular manifestations [18]. As research has progressed, it has become clear that LRG1 does not simply reflect disease status; rather, it may actively regulate inflammation and pathological vascular responses. Studies have shown that LRG1 acts as a key activator of macrophages and induces polarization toward the pro-inflammatory M1 phenotype during the development of AS [12]. This indicates that LRG1 is not merely an external indicator of “inflammation,” but may instead be a functional molecule involved in shaping a pro-inflammatory immune microenvironment. In endothelial cells, LRG1 interacts with endothelial glycoproteins and induces pathological angiogenesis and vascular dysfunction through the TGFβR2-activin A receptor-like type 1 (ACVRL1)-SMAD1/5/8 pathway [19]. Similarly, during the inflammatory phase of wound healing in mice, LRG1 levels are markedly increased, with bone marrow-derived cells serving as the major source of LRG1; LRG1 deficiency impairs immune cell infiltration, re-epithelialization, and angiogenesis, ultimately leading to a significant delay in wound closure [20], which further highlights its close association with vascular responses and tissue repair during inflammation. In other disease models, LRG1 has also been shown to be a key regulator of inflammation and angiogenesis. For example, in mechanistic studies of osteoarthritis, tumor necrosis factor-alpha (TNF-α)-induced LRG1 expression significantly promoted the angiogenesis and migration of mesenchymal stem cells in subchondral bone [21]. Blocking LRG1 may also affect immune responses and the local inflammatory microenvironment. Studies using LRG1-blocking antibodies to inhibit Smad3 suggest that this strategy may enhance immune responses, indicating that LRG1 may influence not only the vascular system itself but also immune regulation [22]. In a preclinical tumor model in which CD8+ T cells targeted previously injected melanoma cells in an antigen-specific manner, LRG1 acted locally at sites of inflammation by modulating the TGF-β signaling pathway; blocking LRG1 with a specific antibody reduced tumor size by 30% and restored vascular function [23], further supporting its characterization as a regulator of pathological vascular abnormalities. Another study showed that in the absence of LRG1, the area occupied by disorganized retinal neovascular growth (cluster-like structures) in diabetic retinopathy was significantly reduced, suggesting that LRG1 is required for pathogenic angiogenesis [24]. Conversely, exogenous LRG1 interfered with the maturation of developing retinal vessels and shifted vascular development toward a dysfunctional phenotype, confirming that LRG1 can act as a pathogenic vascular factor [25]. Collectively, the pathophysiological significance of LRG1 in the vasculature is first reflected in its role as a critical molecular node linking inflammatory responses to vascular abnormalities.

In addition to its roles in inflammation and vascular abnormalities, LRG1 is closely associated with tissue repair, extracellular matrix (ECM) remodeling, and fibrosis-like changes, extending its pathological impact from vascular responses to structural consequences at the tissue level. The upregulation of LRG1 is not a random phenomenon; rather, it can be regarded as an important molecular signal indicating the transition from tissue homeostasis to pathology. Across multiple organs, LRG1 amplifies the canonical TGF-β/Smad2/3 cascade, thereby enhancing fibroblast activation and myofibroblast differentiation in the lung, kidney, liver, skin, eye, and joints, ultimately promoting pathological fibrosis [26]. Its function also appears to be context dependent. For example, in cardiac fibroblasts, LRG1 inhibits fibroblast activation by competing with TGFβ1 for receptor binding, whereas peroxisome proliferator-activated receptor β/δ (PPARβ/δ) and TGFβ1 synergistically regulate LRG1 expression through silencing mediator for retinoid and thyroid hormone receptors (SMRT), suggesting a functional interaction between LRG1 and PPARβ/δ during cardiac fibroblast activation [27]. This suggests that the role of LRG1 in the remodeling process is not merely a mechanical, unidirectional promotion of fibrosis, but is rather contingent upon the specific tissue context and signaling environment. Specifically, regarding tissue repair and remodeling after injury, one study examined the expression pattern of LRG1 following full-thickness cutaneous wounding in mice. Immunohistochemical analysis showed that LRG1 was overexpressed in the epidermis after injury, and this elevated expression persisted throughout the entire repair period, suggesting that LRG1 may be involved in the repair process after epidermal or dermal injury [28]. Another study indicates that exogenous LRG1 can accelerate wound healing by promoting keratinocyte migration in animal models [20]. However, in chronic or abnormal pathological settings, this reparative tendency may shift toward pathological remodeling. For example, one in vitro study demonstrated that in ARPE-19 retinal pigment epithelial cells, LRG1 promotes epithelial–mesenchymal transition in retinal pigment epithelial cells, thereby facilitating subretinal fibrosis, causing loss of previously acquired vision, and even leading to retinal detachment due to traction from fibrous membranes, with severe structural consequences [29]. Tissue remodeling is often accompanied by an imbalance between ECM deposition and degradation, including increased collagen deposition, matrix stiffening, and fibrosis-like changes. LRG1 may indirectly influence ECM remodeling by regulating cytokine networks, growth factor signaling, and changes in cellular phenotype. One study demonstrated that during scar formation, mechanical loading transmitted from the ECM to the membrane of human dermal fibroblasts stimulates FAK phosphorylation and thereby activates ERK. Phosphorylated ERK (p-ERK) then translocates from the cytoplasm to the nucleus and phosphorylates ELK1, leading to ELK1 binding to the *LRG1* promoter region together with cofactors, which initiates transcription and stimulates *LRG1* expression [30]. Another study showed that upregulation of LRG1 is one of the major factors promoting ECM synthesis and may contribute to structural consequences such as synovial stiffness in osteoarthritis [31]. Thus, LRG1 is involved not only in acute injury repair but also in aberrant repair and tissue remodeling under chronic pathological conditions. Its role has expanded beyond the inflammation–vascular axis to include ECM metabolism, phenotypic alterations, and fibrosis-like structural changes.

In the cardiovascular system, vascular tissue remodeling is a complex adaptive process that primarily involves a series of structural and functional changes within the three-layered structure of the vascular wall, including intimal hyperplasia, thickening and thinning of the media, and adventitial fibrosis. The ECM—comprising matrix proteins and their degrading metalloproteinases—constitutes a major component of the vascular microenvironment and undergoes dynamic changes during the process of vascular remodeling [32]. One study suggests that LRG1 reduces expression of the gap junction protein connexin 43 in arteries and capillaries, but not in veins, which may impair communication between endothelial cells and pericytes, thereby leading to vascular dysfunction and affecting vascular remodeling [25]. Moreover, AS is not driven by a single mechanism; rather, it is a complex process promoted jointly by inflammation, endothelial dysfunction, cellular phenotypic switching, ECM remodeling, and vascular remodeling [33]. Inflammation runs through the entire course of AS, from initiation and progression to the development of complications, and both lesion evolution and plaque complications are driven by inflammatory mechanisms [34]. At the same time, phenotypic switching of vascular smooth muscle cells (VSMCs) and endothelial cells is driven by chronic inflammation and, in turn, influences lesion progression [35]. Within this pathological framework, if LRG1 participates simultaneously in the regulation of inflammation, abnormal vascular responses, impaired tissue repair, and vascular remodeling, then its significance in AS may extend beyond that of a general inflammatory marker to involve lesion morphological evolution, changes in plaque structure, and the trajectory of disease progression. In this sense, LRG1 intersects with several core pathological processes in AS: it can regulate endothelial TGF-β signaling and promote abnormal angiogenesis; it can impair pericyte coverage and vascular maturation, thereby affecting the vascular wall microenvironment and basement membrane-associated structures; and it is also linked to inflammatory amplification, ECM remodeling, and the evolution of structural lesions. Therefore, from the perspective of vascular pathophysiology, LRG1 has a theoretical basis for being regarded as a candidate molecule, and potentially even a therapeutic target, relevant to AS.

## 3. Relevant Evidence Regarding LRG1 and AS

### 3.1. Clinical Evidence

Depending on the vascular bed involved, AS can present as a spectrum of clinical phenotypes, including atherosclerotic coronary artery disease (angina pectoris and myocardial infarction), cerebrovascular disease (cerebral infarction and transient ischemic attack), peripheral arterial disease (intermittent claudication), and renovascular hypertension. Therefore, assessment of whether LRG1 is related to AS should not be limited to the plaque itself, but should also consider its clinical relevance across the broader spectrum of atherosclerotic cardiovascular disease. Existing clinical studies suggest that LRG1 is associated with multiple atherosclerotic clinical phenotypes. In a study examining the relationships between plasma LRG1 levels, arterial stiffness, endothelial function, and peripheral arterial disease (PAD), 2058 patients with type 2 diabetes were recruited and stratified according to ankle–brachial index (ABI) into a PAD group (ABI ≤ 0.9), a borderline ABI group (ABI 0.91–0.99), and a normal group (ABI 1.00–1.40). Compared with the borderline and normal groups, patients with PAD had significantly higher LRG1 levels, suggesting that elevated LRG1 is an important predictor of arterial stiffness, endothelial dysfunction, and PAD [36]. In another cohort study assessing cardiovascular disease risk in patients with end-stage renal disease (ESRD), 169 adults receiving chronic hemodialysis were enrolled. In this cohort, the overall prevalence of cardiovascular disease was 27%, with coronary artery disease (CAD) and congestive heart failure (CHF) being the most common conditions, followed by peripheral arterial occlusive disease (PAOD), prior myocardial infarction (MI), and stroke. This study indicated that elevated LRG1 levels are closely associated with systemic inflammation and an increased risk of PAOD and cardiovascular disease in patients with ESRD [37]. In addition, a study investigating novel protein biomarkers associated with CAD in statin-treated patients with familial hypercholesterolemia (FH) compared plasma protein profiles across different stages of atherosclerotic burden. The study identified six plasma proteins that were significantly associated with more advanced stages of atherosclerotic disease in FH, among which LRG1 showed a particularly notable association with CAD staging [38]. Collectively, these studies suggest that LRG1 is not limited to a single disease type in clinical settings; rather, it shows changes consistent with atherosclerotic phenotypes across coronary disease, peripheral vascular disease, and high-risk metabolic and renal populations, indicating broad clinical relevance.

In addition to its association with chronic atherosclerotic phenotypes, LRG1 also appears to have potential value in acute coronary events. Acute coronary syndrome (ACS) and ST-segment elevation myocardial infarction (STEMI) are, in essence, acute clinical events triggered by plaque inflammation, rupture, or erosion. If LRG1 remains elevated or changes dynamically during this phase, this would further support its association with disease activity. One study used proteomic analysis to examine serum samples from 50 patients with ACS and 50 healthy controls in order to identify serum biomarkers associated with ACS pathogenesis. The results showed that alpha-1-acid glycoprotein 1 (AGP1), complement C5, LRG1, and vitronectin (VN) were elevated in ACS, and the authors suggested that these proteins are involved in ACS pathogenesis and may provide signals of ACS occurrence even before classic biomarkers of myocardial necrosis become evident [39]. Another study evaluated the predictive value of plasma LRG1 levels at different time points for major adverse cardiovascular events (MACE) in patients with STEMI. A total of 209 patients with STEMI were enrolled, and plasma LRG1 levels were measured by enzyme-linked immunosorbent assay (ELISA) at admission and on days 1, 7, and 30 after admission. Plasma LRG1 levels were already elevated at admission, increased further on day 1, and then gradually declined, suggesting that the dynamic changes in LRG1 after acute myocardial infarction are consistent with the processes of acute inflammation and tissue injury. The study also found that a plasma LRG1 level > 60 μg/mL on day 7 after admission had good predictive value for MACE in these patients [40]. In addition, in a proteomics-based study of novel serum biomarkers for early myocardial infarction (MI), 538 proteins were quantified, and pregnancy zone protein (PZP), LRG1, and apolipoprotein CI (Apo CI) were found to be upregulated in patients with MI. The authors suggested that inflammation-related LRG1 and PZP may serve as novel serum biomarkers for MI [41]. According to a preprint available in 2026, a multicenter study investigating prediction of ACS events compared plasma LRG1 levels among patients with ACS, chronic coronary syndrome (CCS), and healthy controls. The results indicate that LRG1 levels are significantly higher in patients with ACS compared to both the CCS group or the healthy controls. The study suggests that LRG1 may be a novel, independent biomarker for predicting the risk of cardiovascular events in patients with ACS, while also providing supplementary information regarding the risk of cardiovascular event recurrence within the context of secondary prevention for ACS [42]. Overall, these findings suggest that LRG1 is associated not only with chronic atherosclerotic disease but also with lesion activity and event risk in the acute coronary setting, supporting its potential value for early identification and clinical risk indication.

On this basis, current research has further extended the clinical significance of LRG1 from simple disease association to prognostic assessment and risk stratification. In a cohort study evaluating whether serum LRG1 levels were associated with all-cause mortality, vascular mortality, and MACE in patients undergoing coronary angiography, 695 patients with diagnosed or suspected stable coronary heart disease were randomly selected and followed for 10 years. The results showed that patients with CAD had higher LRG1 levels than those without CAD, and the authors proposed that serum LRG1 may serve as a promising novel predictor of all-cause mortality, vascular mortality, and MACE in patients undergoing coronary angiography [43]. The importance of this study lies in showing that LRG1 may indicate not only the presence of coronary lesions but also the likelihood of future adverse outcomes. Similarly, a prospective study of clinical outcomes in patients with confirmed peripheral arterial disease (PAD) enrolled 295 patients with ultrasound-confirmed PAD and followed them for 10 years. The primary endpoints were all-cause mortality, cardiovascular mortality, and MACE. The study found that elevated LRG1 levels were significantly associated with increased risk for all major endpoints and concluded that LRG1 is a strong independent predictor of 10-year mortality in patients with PAD [44]. As noted above, the study in patients with ESRD also showed that, in addition to its association with CVD and PAOD risk, LRG1 levels were positively correlated with IL-6, high-sensitivity C-reactive protein (hsCRP), and more advanced T-cell differentiation, suggesting that LRG1 may contribute to AS progression by promoting inflammation [37]. Overall, current clinical studies provide preliminary support for an association between LRG1 and AS at three levels: disease presence, indication of acute events, and long-term risk stratification. However, it should also be recognized that current study populations are still mainly limited to the spectrum of atherosclerotic cardiovascular disease, such as CAD, ACS, STEMI, PAD, and high-risk renal disease or FH populations, whereas clinical evidence specifically based on imaging- or pathology-defined “narrow” AS cohorts remains relatively limited. Thus, the clinical evidence should be interpreted mainly as association and prognostic evidence. It supports the clinical relevance of LRG1, but it does not by itself prove that LRG1 directly drives plaque formation, progression, or rupture. The main clinical studies linking LRG1 to atherosclerotic cardiovascular phenotypes are summarized in Table 1.

### 3.2. Evidence from Lesion-Level Studies and Experimental Models

LRG1 is not only abnormally elevated in the systemic circulation but can also accumulate locally within AS lesions. In a study investigating the role of LRG1 in advanced AS and calcification, serial cryosections of the aortic sinuses from atherosclerotic mice were analyzed to assess their lipid content and plaque calcification status. The results revealed that the LRG1 expression colocalized with the stained regions and was mainly distributed around microcalcifications, particularly accumulating in larger punctate calcified areas. To further validate these observations in human specimens, the study also analyzed LRG1 expression in human atherosclerotic plaques obtained via carotid endarterectomy. The results were consistent with those in mice and suggested that LRG1 preferentially localizes to calcified regions of atherosclerotic plaques. These findings indicate that LRG1 is not only present in AS lesions but is more likely to be enriched in complex plaques, particularly in regions with calcific changes [45].

In addition to direct evidence for lesion localization, existing studies also suggest that LRG1 may be associated with plaque severity and changes in the lesional microenvironment. In a machine learning-based study investigating key genes and mitochondrial-related mechanisms associated with the severity of AS, a dataset of atherosclerotic plaques from eight human samples in the GEO database was used to identify genes related to plaque severity. Differential gene analysis (DGA) showed that 4012 differentially expressed genes (DEGs) were identified in the carotid atherosclerotic plaque group, among which 3167 were upregulated; *LRG1* was one of these upregulated genes [46]. More direct experimental evidence comes from a recent study of LRG1 in CAD and experimental AS. This study reported higher plasma LRG1 levels in patients with CAD than in non-CAD controls. LRG1 was also increased in femoral plaques from patients with severe femoral stenosis, and stronger LRG1 staining appeared in regions with CD68, VE-cadherin, and VCAM-1 signals. In *Apoe^−/−^* mice, *Lrg1* deletion reduced atherosclerotic lesion areas and decreased proinflammatory monocytes and macrophage-related cytokines. Anti-LRG1 neutralizing antibody also inhibited LRG1-induced macrophage M1-like polarization and reduced plaque burden in *Apoe^−/−^* mice. Mechanistically, this macrophage response was mainly related to ERK1/2 and JNK signaling. These findings provide direct preclinical evidence that LRG1 may promote AS through macrophage-driven inflammation [12]. These findings support a local association between LRG1 and complex or calcified plaques. However, the localization evidence remains limited. Current data do not fully define which cell types produce LRG1 in human plaques. They also do not clarify its distribution across macrophage-rich regions, neovascular areas, calcified lesions, or rupture-prone sites. Human atherosclerotic plaques contain heterogeneous immune, endothelial, and smooth muscle cell populations, as shown by single-cell transcriptomic studies [47]. Spatial omics strategies may further help link molecular signals with plaque regions and tissue architecture [48]. These studies do not provide direct LRG1-specific spatial validation currently. Rather, they highlight why LRG1-focused validation is needed. Future studies should combine single-cell RNA sequencing, spatial transcriptomics, spatial proteomics, and imaging-based methods, such as multiplex immunofluorescence or RNA in situ hybridization, to clarify the cellular source and spatial pattern of LRG1 in human plaques. The currently available lesion-level and direct experimental evidence linking LRG1 to AS is summarized in Table 2.

## 4. Mechanistic Basis for LRG1 as a Potential Therapeutic Target in AS

**Figure 1:**
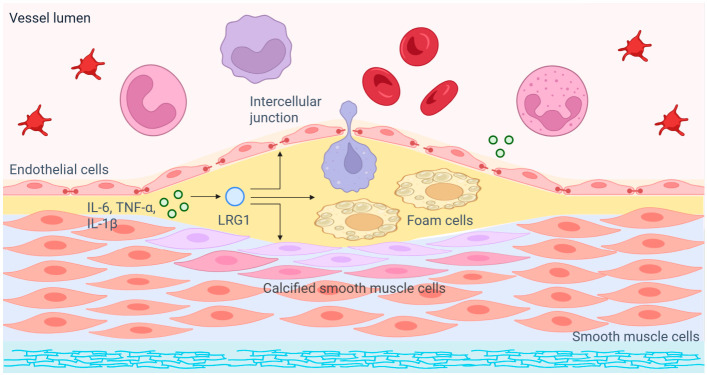
Proposed plaque-centered framework of LRG1 in atherosclerotic plaque evolution.

LRG1 may have therapeutic relevance in AS because its effects appear to converge on several processes that shape plaque evolution. These include chronic inflammation, endothelial dysfunction, abnormal angiogenesis, ECM remodeling, and changes in plaque structure. These processes are not isolated. In a plaque-centered framework, inflammation may first increase LRG1 expression. LRG1 may then influence immune activation, endothelial homeostasis, and ECM remodeling. Introducing this framework can help explain the association between LRG1 and AS and provide more basis for discussing its potential as a therapeutic target. The proposed plaque-centered framework is summarized in Figure 1.

AS is generally considered a disease driven by chronic sterile inflammation. Macrophages are among the major immune cells in AS lesions. Their activation, foam-cell formation, and association with necrotic core expansion are closely related to persistent plaque inflammation and plaque fragility [49,50]. In this inflammatory microenvironment, IL-6 is one of the key mediators closely associated with AS. It not only reflects inflammatory activity but also amplifies the inflammatory response. IL-6 can activate multiple immune cells, including macrophages and T cells. These cells then release additional cytokines such as TNF-α and interleukin-1 (IL-1). This cytokine cascade further sustains plaque inflammation and contributes to the persistence of chronic vascular inflammation [51]. This IL-6-related inflammatory axis may also provide an upstream route for LRG1 induction. Sustained IL-6 signaling, especially through trans-signaling, is associated with endothelial dysfunction, high-risk atherosclerotic plaques, myocardial fibrosis, and adverse remodeling [52,53]. IL-6 can also promote coronary AS progression through the JAK/STAT3 pathway. For example, HIF-1α was reported to aggravate coronary AS by activating the IL-6/JAK1/STAT3 pathway, whereas targeting HIF-1α attenuated lesions by suppressing this inflammatory cascade [54]. Since LRG1 can be induced by IL-6/STAT3 signaling, it may be positioned downstream of inflammatory activation and may further participate in macrophage-driven inflammation. In this sense, LRG1 may contribute to an inflammatory feed-forward loop in AS rather than simply serving as a passive inflammatory marker.

Beyond immune-cell activation, this framework also places LRG1 at the interface between inflammation and endothelial remodeling. The progression of AS plaques also depends on endothelial barrier dysfunction, abnormal intra-plaque neovascularization, and insufficient microvascular maturation [55,56]. Endothelial dysfunction is characterized by impaired vascular tone and homeostasis. It is accompanied by reduced nitric oxide (NO) production, increased oxidative stress, and enhanced inflammatory responses. Once endothelial integrity is compromised, lipoprotein retention, leukocyte adhesion, and VSMC proliferation are promoted. These changes accelerate plaque formation [57]. Endothelial injury can also interact with intraplaque neovascularization. As intraplaque neovessels increase, their immature and leaky structure allows more inflammatory cells to enter the plaque. Upregulated adhesion molecules further enhance this process. These recruited inflammatory cells then release cytokines, growth factors, and angiogenic mediators, which further stimulate neovessel formation. As a result, inflammation and neovascularization form a positive feedback loop that increases plaque complexity [58]. Inflammatory cells can release classical pro-angiogenic cytokines, including IL-6, TNF-α, monocyte chemoattractant protein-1 (MCP-1), and interleukin-8 (IL-8). They may also release other specific factors that promote angiogenesis and aberrant vascular responses [59]. In this context, LRG1 may connect inflammatory activation with endothelial injury and abnormal angiogenesis. It may help transfer inflammatory signals to the vascular wall. Increased LRG1 may disturb endothelial homeostasis and promote immature neovessels. These changes may allow more inflammatory cells to enter plaques. Therefore, LRG1 may contribute to plaque progression and structural complexity.

The formation and changes in stability of AS plaques are accompanied by ECM remodeling. As a primary component of the vascular microenvironment, the ECM undergoes dynamic changes during the process of vascular remodeling and directly participates in both the formation of atherosclerotic plaques and the maintenance of their stability [60,61]. VSMCs are one of the predominant cell types in normal arteries. However, in response to atherosclerotic stimuli, VSMCs respond to changes in blood flow and the microenvironment by downregulating contractile markers and altering their phenotype [62]. These changes provide the structural basis for linking endothelial injury and abnormal angiogenesis to plaque remodeling. Fibrotic plaques in women show smooth muscle cell-driven ECM remodeling, active TGF-β signaling, and endothelial-to-mesenchymal transition. TGF-β can induce endothelial-to-mesenchymal transition in endothelial cells. It can also promote ECM remodeling in VSMCs [63]. In rat aortic smooth muscle cells, exogenous TGF-β1 enhances smooth muscle cell calcification over time. Other pro-calcific stimuli may suppress TGF-β1 expression during the early, intermediate, and mature stages of calcification, but this inhibitory effect gradually weakens. These findings suggest that TGF-β1 is also involved in vascular inflammation and calcification [64]. The strength and specificity of TGF-β signaling depend on cell-surface coreceptors and secreted molecules. These molecules interact with TGF-β and its receptors in a cell-specific manner. Therefore, factors that amplify or redirect TGF-β signaling may affect plaque structural evolution. As a secreted glycoprotein, LRG1 can promote the binding of TGF-β to endothelial glycoproteins and TGF-β receptors, thereby enhancing TGF-β signaling [65]. In a mouse model of diabetic nephropathy, LRG1 has also been identified as a novel pro-angiogenic gene. Studies have shown that LRG1 enhances angiogenesis and endothelial proliferation in diabetic mice by augmenting endothelial TGF-β/activin receptor-like kinase 1 (ALK1) signaling [66].

Thus, LRG1 may participate in plaque morphological evolution and structural remodeling by modulating TGF-β signaling, ECM metabolism, smooth muscle cell phenotype, and calcification. In the plaque-centered framework proposed above, LRG1 may therefore connect inflammation-induced endothelial injury with later matrix remodeling and plaque structural changes. These processes may reinforce each other and contribute to plaque progression and instability. This provides an important mechanistic basis for considering LRG1 as a potential therapeutic target in AS.

## 5. Feasibility of LRG1 as a Therapeutic Target in AS

Viewed through the lens of general principles for target screening, LRG1 possesses several fundamental characteristics favorable for drug development. First, LRG1 is a secreted protein located predominantly in the extracellular space, which in theory makes it more amenable to direct targeting by antibodies, ligand-modified nanodelivery systems, or targeted degradation strategies than intracellular transcription factors or complex multiprotein assemblies. Second, current clinical observations suggest that LRG1 is associated not only with coronary lesions, PAD, adverse events after STEMI, and long-term mortality risk within the spectrum of atherosclerotic cardiovascular disease [40,43], but also with heavier vascular disease burden in high-risk populations with renal disease and diabetes [36,37], implying that it is not only detectable but also potentially useful for disease stratification [67]. In other words, LRG1 is not merely a molecule discussed in laboratory settings; it is measurable with relative stability in clinical samples and has potential risk-indicating value. In addition, LRG1 is linked not to a single isolated terminal event, but rather to the intersection of multiple pathological processes, including inflammatory amplification, endothelial imbalance, abnormal vascular responses, and tissue remodeling. Therefore, in theory, it is more likely to influence key nodes in the course of AS rather than simply serving as a marker that passively rises with disease activity, and thus has a basic rationale for consideration as a therapeutic target.

From the perspective of true druggability, LRG1 has already accumulated substantial cross-disease empirical support. At present, the most mature line of work comes from ophthalmology, where a humanized antibody targeting LRG1 was developed to inhibit angiogenesis and reduce retinal vascular leakage. This antibody was able to suppress pathological angiogenesis and vascular leakage in both in vitro and in vivo mouse models, and no toxicity or inflammatory response was detected in vivo. These findings suggest that LRG1 can be functionally blocked and may represent a novel candidate therapeutic target [68]. Another study analyzed the structural interaction between the LRG1 epitope and the Fab fragment of Magacizumab, a humanized IgG4 antibody that functionally blocks LRG1, and identified its specific binding mode and the key residues involved in LRG1 recognition. Based on these findings, a series of mutations were proposed that hold promise for enhancing antibody affinity. These mutations can be introduced into Magacizumab to boost its affinity for LRG1, thereby potentially enabling a reduction in both the dosage and frequency of administration for patients [69]. Although this work is rooted in structural biology, it indicates that antibody development against LRG1 has moved beyond the question of simple feasibility and entered a more advanced stage focused on optimizing binding and improving dosing characteristics. In addition to antibody blockade, targeted degradation strategies directed against LRG1 have also emerged. Studies have revealed that LRG1 is significantly upregulated in renal fibrosis, potentially exacerbating the disease by enhancing the TGF-β–Smad3-dependent signaling pathway. Building upon this insight, researchers utilized a previously developed LRG1-targeting peptide (to recruit LRG1) in conjunction with lenalidomide (to activate E3 ubiquitin ligase) to construct an advanced proteolysis-targeting chimera (ET TAC-2) specifically designed to induce the degradation of LRG1. Cellular degradation assays confirmed that ET TAC-2 effectively degrades LRG1 via a proteasome-dependent mechanism. In vitro and in vivo antifibrotic studies demonstrated that ET TAC-2 can effectively induce the degradation of LRG1 within fibrotic kidneys. This intervention effectively inhibited the TGF-β–Smad3 signaling pathway and reduced the secretion of fibrosis-associated proteins, thereby slowing the progression of renal fibrosis. Consequently, ET TAC-2 holds promise as a potential therapeutic candidate for targeted intervention against LRG1 [70]. In a corneal neovascularization (CNV) model, LRG1 was similarly demonstrated to be significantly upregulated, driving neovascularization through the TGF-β-Smad signaling pathway. Researchers employed Proteolysis-Targeting Chimera (PROTAC) technology—specifically utilizing the newly developed compound ET TAC-2—to selectively degrade LRG1 in a mouse model of alkali-burn-induced injury. In vitro experiments demonstrated that ET TAC-2 effectively degraded LRG1 in a time- and dose-dependent manner; in vivo studies revealed that ET TAC-2 significantly reduced LRG1 levels within the CNV lesions and inhibited the release of angiogenic factors by suppressing the TGF-β-Smad1/5/9 pathway, thereby slowing the progression of CNV. This study positions ET TAC-2 as a potential therapeutic candidate for the treatment of CNV [71]. These studies show not only that LRG1 can be degraded, but also that this molecule is suitable for entry into relatively cutting-edge therapeutic modalities, including PROTAC-based targeted degradation. Beyond biologics and degradation technologies, LRG1 also shows considerable potential in the realm of targeted drug delivery. One study leveraged the characteristic upregulation of LRG1 protein expression during the progression of renal fibrosis to develop a novel drug delivery system. The researchers designed a nanocarrier, designated DEN NM, which preferentially targets fibrotic kidneys by exploiting the high affinity of a modified ET peptide for LRG1. Upon internalization by damaged renal cells, DEN NM releases its encapsulated payload—Nintedanib—triggered by the activation of active caspase-3 protease. Nintedanib effectively reduces the expression levels of the ECM and halts the progression of renal fibrosis by inhibiting the TGF-β–Smad2/3 signaling pathway. Both in vitro and in vivo experimental results have validated the anti-fibrotic efficacy of DEN NM, underscoring LRG1’s potential as a molecular anchor for targeted drug delivery within the kidney [72]. In oncology, another study developed a nanomedicine, OCE NM, centered on a camptothecin (CPT)-ET conjugate. By targeting LRG1-high lesions, this system preferentially delivers CPT to LRG1-overexpressing colorectal cancer tissue and combines it with the potent poly (ADP-ribose) polymerase (PARP) inhibitor olaparib to enhance DNA damage and inhibit repair, thereby promoting tumor-cell apoptosis. These findings further indicate that the significance of LRG1 is not limited to its role as a pathological molecule; it can also serve as a recognition site for precision drug delivery, thereby improving local drug-delivery efficiency and underscoring the therapeutic potential of combining CPT with olaparib in colorectal cancer treatment [73]. From the perspective of drug development, if a target can not only be blocked but also utilized for targeted delivery, its translational value is often significantly higher.

The core rationale for mapping these cross-disease insights onto AS lies in the fact that the AS plaque itself constitutes a complex microenvironment, collectively defined by macrophage-endothelial cell crosstalk, pathological neovascularization, ECM remodeling, and the amplification of local inflammation [74,75]. Due to the uneven distribution of conventional drugs, dose-limiting toxicities, and low bioavailability at lesion sites, they often demonstrate limited efficacy in achieving significant regression or reversal of atherosclerotic plaques. Moreover, the microenvironment of AS plaques constitutes a formidable biological barrier that significantly diminishes the therapeutic efficacy of interventions. As a result, increasing attention has been directed toward novel delivery platforms capable of enhancing local therapeutic effects, improving lesion accumulation, and reducing off-target reactions, including polymeric, lipid-based, and inorganic nanoparticles. These platforms have shown growing promise in the prevention and treatment of AS [76]. In light of the fact that multiple technological approaches centered on LRG1—including antibody blockade, targeted degradation, and drug-delivery anchoring—have already emerged in other disease models, and given that AS is itself a disease context highly responsive to precise local delivery and microenvironment-targeted intervention, these cross-disease experiences are not entirely non-transferable. To present these translational strategies more clearly, the main LRG1-targeted approaches and their AS-related implications are summarized in Table 3.

However, the feasibility of LRG1 as a therapeutic target for AS must still be defined with caution. The currently available evidence regarding direct therapeutic intervention stems primarily from indications other than AS. Whether in the form of antibody development in ophthalmology, targeted degradation studies in renal fibrosis and corneal neovascularization, or targeted drug-delivery platforms in oncology and nephrology, these efforts demonstrate that LRG1 is druggable, but they cannot be taken as direct proof that its indication in AS has already been validated. Furthermore, there are currently no mature drug development pipelines specifically dedicated to targeting LRG1 for AS. This implies that direct answers remain elusive regarding the optimal stage for intervention within coronary arteries or atherosclerotic plaques, the most suitable route of administration, and the long-term safety and efficacy of such therapies. Moreover, AS is an inherently highly heterogeneous disease in which the primary pathological drivers vary significantly among patients: in some, the disease is predominantly lipid-driven; in others, it is characterized by persistent inflammatory amplification; while in yet others, abnormal vascularization and fibrotic remodeling are the most prominent features. Consequently, even if LRG1 eventually enters the clinical translation pathway for AS, it is highly unlikely to serve as a universal therapeutic target applicable to all patients. Instead, it is far more likely to be suitable for specific stages of disease progression and distinct phenotypes—particularly for patient subgroups in whom endothelial injury, abnormal microvasculature, and remodeling phenotypes are especially pronounced. Therefore, a more prudent assessment at this juncture is not that LRG1 is already a fully validated therapeutic target for AS, but rather that the existing evidence provides a compelling rationale and translational insights supporting its potential as a future therapeutic target for the disease.

## 6. Discussion

LRG1 has gradually emerged as a molecule of interest in vascular pathophysiology. In the background of AS, current evidence suggests that LRG1 is not only associated with systemic inflammatory status, but also with plaque-related processes. Some clinical studies have linked elevated LRG1 levels with atherosclerotic cardiovascular phenotypes and adverse outcomes. At the lesion level, LRG1 has been associated with plaque calcification. Experimental studies further suggest that it may affect macrophage activation and endothelial function. Its possible roles in abnormal angiogenesis and ECM remodeling also deserve attention. These findings indicate that LRG1 may have relevance beyond its role as a circulating biomarker. It may also participate in the local pathological changes that accompany plaque progression.

The potential importance of LRG1 in AS may lie in its position between inflammation, vascular injury, and structural remodeling. Inflammatory signals, especially the IL-6/STAT3 axis, may increase LRG1 expression. Increased LRG1 may then affect macrophage activation, endothelial homeostasis, and immature neovascularization. Through TGF-β-related signaling, it may also influence ECM remodeling, smooth muscle cell phenotype, calcification, and plaque structural change. This plaque-centered view may also explain the possible added value of LRG1 compared with established biomarkers. Lipid markers mainly reflect lipid-related risk. Inflammatory markers such as hsCRP or IL-6 mainly indicate systemic inflammatory burden. LRG1 should not be viewed as a replacement for these markers. Its potential value may lie in its closer relationship with plaque remodeling, including endothelial injury, abnormal angiogenesis, ECM remodeling, and calcification. It supports the idea that LRG1 may serve as a complementary biomarker or candidate therapeutic target, rather than only a general disease-associated marker.

There are several challenges that should be acknowledged. Clinical studies have repeatedly reported a relationship between LRG1 and atherosclerotic cardiovascular phenotypes. These include CAD, ACS/STEMI, PAD, ESRD-related cardiovascular risk, familial hypercholesterolemia, and long-term vascular outcomes [36,37,38,39,40,41,42,43,44]. Such findings support the clinical relevance of LRG1. However, they remain largely associative. They cannot determine whether LRG1 directly promotes plaque formation, progression, or rupture. Evidence from plaque-level studies is also still incomplete. LRG1 has been found to accumulate in complicated plaques and calcified regions. Transcriptomic analyses also suggest increased LRG1 expression in atherosclerotic plaques [45,46]. Even so, its cellular source in human lesions is not fully defined. Its spatial distribution and stage-specific expression also remain unclear. This is an important gap, because recent single-cell and spatial transcriptomic studies have shown marked heterogeneity among immune cells, endothelial cells, and smooth muscle cells in human plaques. These cell states are also organized differently across plaque regions [77,78]. LRG1-specific spatial validation in human AS plaques therefore remains insufficient. Third, although IL-6/STAT3 and TGF-β-related pathways provide plausible mechanistic links, many of these links are inferred from different disease models or non-AS vascular contexts [18,65,66]. Their direct relevance to AS still requires careful validation. Finally, therapeutic studies targeting LRG1 in AS are still insufficient. Current interventional evidence mainly comes from ocular disease, renal fibrosis, corneal neovascularization, tumor angiogenesis, and other non-AS models [68,69,70,71,72,73]. Therefore, LRG1 should not yet be regarded as a fully validated therapeutic target for AS.

Future studies should therefore move from association to direct validation. The current evidence suggests two relatively clear directions in AS. One is the inflammatory axis, in which LRG1 promotes macrophage M1-like polarization and accelerates atherosclerosis progression. The other is the plaque-structure axis, in which LRG1 accumulates in complicated plaques and may contribute to vascular calcification [12,45]. These findings provide a starting point, but they are still not enough to define LRG1 as a validated therapeutic target in AS. Further studies should test whether blocking or deleting LRG1 can improve plaque stability, reduce macrophage inflammation, limit abnormal neovascularization, or modify calcification in *Apoe^−/−^* or *Ldlr^−/−^* models. Human plaque studies should also clarify whether LRG1 is enriched in macrophage-rich regions, immature neovessels, calcified areas, or rupture-prone sites. Single-cell and spatial approaches may be useful for this purpose [77,78]. At the translational level, anti-LRG1 antibodies, targeted degradation, and LRG1-guided delivery systems have shown promise in many diseases. Their value in AS has not yet been validated. Thus, LRG1 should currently be regarded as a promising but still unproven candidate target in AS. Its real value will depend on stronger plaque-level, mechanistic, and interventional evidence.

## Figures and Tables

**Table 1:** Clinical association evidence linking circulating LRG1 to atherosclerotic cardiovascular phenotypes.

Ref.	Clinical Setting	Study Material/ Design	Main LRG1-Related Finding	Evidence Type/Interpretation
[36]	Type 2 diabetes with peripheral arterial disease	Plasma LRG1; patients stratified by ankle–brachial index	Higher LRG1 levels were observed in patients with PAD and were associated with arterial stiffness and endothelial dysfunction	Clinical association; possible link with peripheral vascular disease and endothelial impairment
[37]	End-stage renal disease with cardiovascular disease risk	Plasma LRG1 in chronic hemodialysis patients	Elevated LRG1 was associated with systemic inflammation, peripheral arterial occlusive disease, and cardiovascular disease risk	Association evidence in a high-risk renal population
[38]	Familial hypercholesterolemia with different stages of CAD burden	Plasma protein profiling	LRG1 was among proteins associated with more advanced atherosclerotic disease stages	Association with coronary atherosclerotic burden
[39]	Acute coronary syndrome	Serum proteomic analysis in ACS patients and healthy controls	LRG1 was elevated in ACS together with other inflammation- and complement-related proteins	Possible association with acute coronary events
[40]	ST-elevation myocardial infarction	Plasma LRG1 measured at admission and after admission	LRG1 increased dynamically after STEMI; day-7 LRG1 showed predictive value for MACE	Prognostic evidence after acute myocardial infarction
[41]	Myocardial infarction	Proteomics-based serum biomarker screening	LRG1 was upregulated in patients with MI	Possible serum biomarker evidence in MI
[42]	ACS, chronic coronary syndrome, and healthy controls	Multicenter comparison; preprint evidence	LRG1 levels were higher in ACS than in CCS or healthy controls	Possible supplementary value for ACS risk prediction; requires further validation
[43]	Patients undergoing coronary angiography	Serum LRG1; 10-year follow-up	Higher LRG1 was associated with CAD and predicted all-cause mortality, vascular mortality, and MACE	Long-term prognostic evidence in coronary disease
[44]	Ultrasound-confirmed PAD	Serum LRG1; 10-year follow-up	Elevated LRG1 was associated with all-cause mortality, cardiovascular mortality, and MACE	Long-term prognostic evidence in PAD

Note: These studies mainly provide clinical association or prognostic evidence.

**Table 2:** Lesion-level and direct experimental evidence linking LRG1 to AS.

Ref.	Evidence Type	Model/Source	Main LRG1-Related Finding	Interpretation
[45]	Lesion-level localization	Atherosclerotic mouse aortic sinus and human carotid endarterectomy plaques	LRG1 was detected in AS lesions and preferentially accumulated around microcalcifications and calcified plaque regions	Supports a local association between LRG1 and complicated or calcified plaques
[45]	Functional plaque-related evidence	Experimental calcification-related analyses	LRG1 was linked to vascular calcification-related changes	Suggests that LRG1 may participate in plaque structural evolution, especially calcification
[46]	Plaque transcriptomic evidence	Human carotid atherosclerotic plaque dataset analyzed by machine learning and differential gene analysis	*LRG1* was among the upregulated genes in carotid atherosclerotic plaques	Suggests an association between LRG1 expression, plaque severity, and the lesional microenvironment
[12]	Direct preclinical AS evidence	AS experimental model and macrophage-related analyses	LRG1 promoted macrophage M1-like polarization and accelerated AS progression	Provides direct experimental support for the inflammatory role of LRG1 in AS
[12]	Therapeutic intervention evidence in AS model	*Lrg1* knockout or anti-LRG1 neutralizing antibody intervention in experimental AS	Genetic or antibody-based inhibition of LRG1 slowed AS progression	Supports LRG1 as a candidate therapeutic target in experimental AS, but clinical validation is still lacking

Note: This table summarizes direct AS-related evidence. Clinical association studies are listed separately in Table 1.

**Table 3:** Translational roadmap of LRG1-targeted strategies and their potential relevance to AS.

Therapeutic Direction	Representative Evidence	What This Strategy Shows	Possible Relevance to AS	Key Unresolved Issue	Ref.
Extracellular LRG1 blockade	Humanized anti-LRG1 antibody in ocular pathological angiogenesis and retinal vascular leakage	LRG1 can be functionally blocked at the extracellular level; vascular leakage and pathological angiogenesis can be reduced	Supports the feasibility of direct LRG1 blockade, especially for vascular injury and abnormal neovascular responses	Not validated in AS plaques; optimal dose, route, timing, and long-term vascular safety remain unclear	[68]
Antibody optimization	Structural analysis of Magacizumab binding to LRG1	LRG1-antibody recognition can be structurally defined; affinity-improving mutations may be designed	Provides a basis for developing more refined LRG1-targeted antibodies	Structural evidence does not equal therapeutic efficacy in AS	[69]
Targeted LRG1 degradation in fibrosis	ET TAC-2-mediated LRG1 degradation in renal fibrosis	LRG1 can be degraded by a targeted degradation strategy; TGF-β-Smad3-related fibrosis can be attenuated	Suggests that LRG1 is not only blockable but also degradable, which may be relevant to plaque remodeling and fibrosis-like changes	Evidence comes from kidney fibrosis; arterial-wall delivery and plaque selectivity remain untested	[70]
Targeted LRG1 degradation in pathological angiogenesis	PROTAC-based LRG1 degradation in corneal neovascularization	LRG1 degradation can suppress angiogenic signaling and reduce neovascularization	Offers a possible conceptual strategy for LRG1-related intraplaque neovascularization	Ocular neovascularization differs from plaque neovessels; AS-specific validation is lacking	[71]
LRG1-guided drug delivery in remodeling tissue	LRG1-targeted nintedanib nanodelivery in renal fibrosis	LRG1 can serve as a molecular anchor for targeted drug delivery in fibrotic tissue	Suggests a possible delivery concept for remodeling-rich atherosclerotic plaques	Kidney-targeted delivery cannot be directly extrapolated to plaques; penetration into AS lesions remains unknown	[72]
LRG1-high lesion targeting	LRG1-targeted camptothecin nanomicelles combined with olaparib in colorectal cancer	LRG1-high lesions can be used for preferential nanomedicine delivery	Supports the idea that LRG1 may act as a lesion-recognition signal	Tumor microenvironment differs substantially from AS plaques; relevance to AS is indirect	[73]
Plaque microenvironment-targeted delivery context	Nanoparticle-based delivery platforms for AS plaques	Local delivery may improve lesion accumulation and reduce off-target exposure	Provides a rationale for testing LRG1-directed delivery in AS	Current AS nanodelivery evidence is not LRG1-specific	[74,75,76]

Note: This table summarizes translational strategies rather than direct clinical evidence in AS.

## Data Availability

No new data were created or analyzed in this study. Data sharing is not applicable to this article.

## Abbreviations

The following abbreviations are used in this manuscript:

AS	atherosclerosis
LRG1	leucine-rich alpha-2 glycoprotein 1
TNF-α	tumor necrosis factor-alpha
TGF-β	transforming growth factor-beta
ECM	extracellular matrix
CAD	coronary artery disease
PAD	peripheral artery disease
ACS	acute coronary syndrome
STEMI	ST-segment elevation myocardial infarction
ESRD	end-stage renal disease
MACE	major adverse cardiovascular events
CVD	cardiovascular disease
IL-6	interleukin-6
VSMCs	vascular smooth muscle cells
STAT3	signal transducer and activator of transcription 3
